# Diagnostic Advances in Childhood Tuberculosis—Improving Specimen Collection and Yield of Microbiological Diagnosis for Intrathoracic Tuberculosis

**DOI:** 10.3390/pathogens11040389

**Published:** 2022-03-23

**Authors:** Eric Wobudeya, Maryline Bonnet, Elisabetta Ghimenton Walters, Pamela Nabeta, Rinn Song, Wilfred Murithi, Walter Mchembere, Bunnet Dim, Jean-Voisin Taguebue, Joanna Orne-Gliemann, Mark P. Nicol, Olivier Marcy

**Affiliations:** 1 Makerere University-Johns Hopkins University Research Collaboration (MU-JHU) Care Ltd., Kampala P.O. Box 7072, Uganda; ewobudeya@mujhu.org; 2 University of Montpellier, IRD, INSERM, TransVIH MI, 34090 Montpellier, France; maryline.bonnet@ird.fr; 3 Desmond Tutu TB Centre, Department of Paediatrics and Child Health, Stellenbosch University, Cape Town 8000, South Africa; ewal@sun.ac.za; 4 Great North Children’s Hospital, Newcastle-upon-Tyne Hospitals Trust, Newcastle Upon Tyne NE1 4LP, UK; 5 FIND, Chemin des Mines 9, 1202 Geneva, Switzerland; pamela.nabeta@finddx.org; 6 Oxford Vaccine Group, Department of Paediatrics, University of Oxford, Oxford OX 7LEe, UK; rinn.song@paediatrics.ox.ac.uk; 7 Center for Respiratory Disease Research, Kenya Medical Research Institute, Nairobi 00100, Kenya; wbmurithi@gmail.com; 8 Center for Global Health Research, Kenya Medical Research Institute, Kisumu 40100, Kenya; wmchembere@mgic.umaryland.edu; 9 Epidemiology and Public Health Unit, Pasteur Institute in Cambodia, Phnom Penh 12201, Cambodia; dbunnet@pasteur-kh.org; 10 Mother and Child Center, Chantal Biya Foundation, Yaoundé P.O. Box 1936, Cameroon; jvoisintag@yahoo.fr; 11 University of Bordeaux, National Institute for Health and Medical Research (INSERM), Research Institute for Sustainable Development (IRD), Bordeaux Population Health Research Centre, Team GHiGS, 33000 Bordeaux, France; joanna.orne-gliemann@u-bordeaux.fr; 12 Division of Infection and Immunity, School of Biomedical Sciences, University of Western Australia, Perth, WA 6009, Australia; mark.nicol@uwa.edu.au; 13 Division of Medical Microbiology and Institute for Infectious Diseases and Molecular Medicine, University of Cape Town, Cape Town 7701, South Africa

**Keywords:** tuberculosis, children, diagnosis, microbiological tests, sample collection methods

## Abstract

There is no microbiological gold standard for childhood tuberculosis (TB) diagnosis. The paucibacillary nature of the disease, challenges in sample collection in young children, and the limitations of currently available microbiological tests restrict microbiological confirmation of intrathoracic TB to the minority of children. Recent WHO guidelines recommend the use of novel rapid molecular assays as initial diagnostic tests for TB and endorse alternative sample collection methods for children. However, the uptake of these tools in high-endemic settings remains low. In this review, we appraise historic and new microbiological tests and sample collection techniques that can be used for the diagnosis of intrathoracic TB in children. We explore challenges and possible ways to improve diagnostic yield despite limitations, and identify research gaps to address in order to improve the microbiological diagnosis of intrathoracic TB in children.

## 1. Introduction

In 2020, tuberculosis (TB) in children accounted for 12% of the global TB burden and 16% of all TB deaths. Most deaths occur in children who do not access TB treatment: in 2020, only 41% of children with TB received treatment [1,2]. A key barrier towards accessing timely treatment in children remains a TB diagnosis. Intrathoracic TB is the most common form of TB in children, occurring in >75% of children treated for TB; yet it remains challenging to clinically diagnose and microbiologically confirm [3,4]. This is partly due to the non-specific presentation of the disease in children, and partly due to the low sensitivity of available molecular tests and mycobacterial culture to detect mostly paucibacillary forms of the disease. In addition, specimen collection for microbiological testing is challenging, particularly in young children who are often unable to spontaneously expectorate sputum, the standard sample collected to detect TB in adults [5,6].

In resource-limited settings, sample collection and testing are further constrained by a lack of trained healthcare workers, equipment, and in-patient paediatric facilities. In the majority of children, TB diagnosis, therefore, relies on clinical evaluation, which is often challenged by limited healthcare worker skills in the interpretation of clinical findings and chest radiography (CXR), and poor access to CXR.

Globally, advances in TB diagnostics in children remain slow, largely due to the difficulties in evaluating new tests in the absence of an adequately sensitive gold standard. Recent WHO guidelines recommend the use of novel moderate complexity rapid molecular assays as initial diagnostic tests and endorse alternative sample collection methods for children [7,8,9]. However, the uptake of these tools in high-endemic settings remains low [10].

This paper aims to review microbiological tests and sample collection methods that can be used in children for the diagnosis of intrathoracic TB, to explore challenges and possible ways to improve the diagnostic yield despite the known limitations. It also aims to encourage research and innovation in sample collection and test development to improve microbiological diagnosis of intrathoracic TB in children.

## 2. Microbiological Tests

Diagnostic yield can be defined as the total number of cases detected using a specific testing strategy. The diagnostic yield of testing strategies for intrathoracic TB in children depends on the intrinsic diagnostic accuracy of laboratory tests, the number and quality of specimens collected (which in turn rely on experienced personnel), reliable and rapid specimen transport, and access to high-quality laboratory systems. Furthermore, intrathoracic TB in children comprises a broad spectrum of disease manifestations, which are associated with variable recovery of bacilli from secretions [6,11]. While cavitary disease (including TB bronchopneumonia, cavitating Ghon focus, or adult-type disease) typically results in secretions with high bacillary concentrations, other manifestations may have very low bacillary loads, such as the most common form of intrathoracic TB in children, primary lymph node disease [12].

Currently, the performance of available diagnostic tests is fit for TB diagnosis in adults, which is typically characterised by a high bacterial load in the sputum. However, all have limited sensitivity to detect paucibacillary TB, due to inherent technological constraints. Despite recent advances and improvements in bacteriological tests, their performance in childhood TB remains below the expectations of the WHO-endorsed target product profile (TPP) for TB diagnosis [13].

### 2.1. Acid-Fast Staining and Smear Microscopy

Smear microscopy is the oldest microbiological test for TB. Simple and inexpensive, it has been the cornerstone of the WHO DOTS (Directly Observed Treatment, Short-course) TB control strategy, relying on the direct visualisation of the acid-fast bacilli using conventional microscopy based on Ziehl–Neelsen staining or fluorochrome staining with standard/light-emitting diodes (LED) fluorescence microscopy [14] (Table 1). Due to its high limit of detection (LOD) and the low bacillary load in children’s respiratory secretions, the sensitivity of smear microscopy is low, except for older children who present with adult-type cavitary disease (Table 1) [15]. Overall, sputum smear microscopy is positive in only 10% to 15% of children with probable TB [16]. Smear microscopy has been superseded by molecular WHO-recommended rapid diagnostic tests (mWRD) for first-line TB diagnosis in children, but microscopy often remains the only accessible diagnostic test at the primary health care level in many resource-limited settings [8,17].

### 2.2. Mycobacterial Culture and Identification

Mycobacterial culture, which has a LOD of approximately 10 to 100 cfu/mL, is considered the reference standard for TB diagnosis in adults [18]. Viable mycobacteria are cultured on solid or liquid media after sample decontamination, followed by species identification to distinguish *Mycobacterium tuberculosis complex* (MTBc) from *Non-tuberculosis Mycobacteria* (NTM) [18] (Table 1). The automated Mycobacterial Growth Indicator Tube (Bactec MGIT, Becton Dickinson Microbiology Systems, Cockeysville, MD, USA), has higher sensitivity, a shorter time to results, and improved reproducibility compared to culture on solid media such as Löwenstein–Jensen (LJ) [19]. Culture yield is usually low (30% to 40%) in children with intrathoracic TB, although certain disease manifestations are associated with much higher diagnostic yields [11,20,21,22,23]. Classical decontamination methods used on samples prior to culture are known to kill up to 30% of the MTBc population and may be too harsh for paediatric paucibacillary specimens and result in a loss of viable organisms for culture [24]. In addition, enrichment of the MGIT culture medium has been shown to enhance the growth of MTB bacilli from gastric aspirates and other paediatric specimens, resulting in a higher detection and shorter time to culture positivity [25]. Methods to enhance the viability and growth of bacilli from culture are highly relevant for the diagnosis of paediatric TB and should be further evaluated.

Access to mycobacterial culture remains very limited in low-income settings due to the level of infrastructure and technical expertise required [26]. Beyond access, waiting for culture confirmation would result in treatment delay and a high risk of progressing to severe forms of TB in children. TB treatment is therefore commonly started empirically. Although this is not an inappropriate practice, it certainly highlights the lack of confidence of healthcare professionals in the diagnostic tools available for TB confirmation and further compounds the lack of motivation in attempting to confirm TB diagnosis in children.

### 2.3. Molecular Tests

Nucleic acid amplification tests (NAATs) are attractive alternatives to culture as they detect bacterial DNA rather than live bacilli and therefore may not be as prone to loss of bacilli from the transport of specimens or laboratory processes. NAATs are generally rapid assays and do not have a requirement for biosafety level 3 laboratories, as bacteria are deactivated and lysed before testing and therefore do not pose the same infection risk as for culture. Among NAATs, the Xpert MTB/RIF (Xpert; Cepheid, Sunnyvale, CA, USA) assay, was a major recent breakthrough in the TB diagnostic landscape and a major advance over prior in-house and commercial NAATs which had heterogeneous diagnostic performances [27,28]. Xpert uses a closed automated system for sample preparation, amplification and real-time (RT) PCR detection of both MTBc and rifampin (RIF) resistance in 2 h. It was endorsed by WHO in 2013 for use in all children following a meta-analysis of the first paediatric studies showing pooled sensitivities for culture-confirmed TB of 62% in expectorated or induced sputum and 66% in gastric aspirates, with very high specificity (98%) [4,17]. The assay requires very little manipulation before testing. In addition, as PCR amplification and detection are integrated into a closed unit, and as the reagent used for sputum liquefaction is tuberculocidal, Xpert has a very favourable biosafety profile compared to smear microscopy slide preparation [29]. However, despite Xpert providing more rapid results compared to culture, and having better sensitivity and specificity than smear microscopy, it has had a limited impact on childhood TB diagnosis overall, due to low overall confirmation rates, and the ongoing challenge of obtaining adequate specimens from children [30,31,32].

Xpert MTB/RIF Ultra (Ultra) was developed by Cepheid as the next-generation assay to address the limited sensitivity of Xpert on smear-negative specimens. It uses the same platform as the Xpert test and employs improved PCR technology and two different amplification targets, resulting in greater sensitivity and reproducibility of MTBc and RIF resistance detection [7]. At very low bacillary DNA concentrations, the Ultra assay provides semiquantitative positive “trace call” results. Not infrequently, such trace calls are associated with negative cultures if performed on the same samples, especially among recently treated patients, which is not commonly the case in children, raising the possibility of false-positive results [33]. However, as paucibacillary TB is most common in high-risk patient groups, including children, HIV-infected individuals, and in extra-thoracic TB (such as TB meningitis), WHO has recommended that trace calls be regarded as microbiological confirmation in these high-risk groups [7,34]. Recently, TB symptoms, extra-pulmonary TB, and no prior TB have been identified as independently associated with culture-positive TB in adults and children with trace calls [35].

The mWRD Truenat™ MTB and MTB Plus are new, low-complexity rapid assays developed by Molbio Diagnostics, Goa, India. They employ chip-based Real-Time (RT) micro-PCR on automated and fully integrated, battery-operated devices for the semiquantitative detection of MTBc directly from sputum specimens [36]. The assays are designed for use in peripheral laboratories with low infrastructure requirements by minimally trained technicians. In case of positive MTB or MTB Plus assay results, the Truenat MTB-RIF assay can be run on an aliquot of extracted DNA to detect mutations associated with RIF resistance [7]. No paediatric data are currently available for the Truenat platform, but given the performance on adult samples, this assay has been endorsed by WHO for use in children as well [8].

A third mWRD is the TB-LAMP assay (Eiken Chemical Company, Tokyo, Japan). It is based on a loop-mediated isothermal amplification reaction, in which target DNA is amplified at a fixed temperature (in contrast to the PCR, which requires a thermocycler) and is visually detected using an ultraviolet lamp, directly in the reaction tubes. This method requires basic equipment which can be implemented at low levels of the laboratory network. However, this method does not allow for the detection of mutations associated with drug resistance. One study in children reported 84% sensitivity and 96% specificity against a microbiological reference standard [37]. This assay is currently endorsed by WHO for use in adults only [8].

### 2.4. Assays Based on the Detection of Urinary Lipoarabinomannan

The mycobacterial cell-wall glycolipid lipoarabinomannan (LAM) is released by metabolically active and dying organisms, filtered by the kidneys, and can be detected in the urine [38]. The urine lateral flow lipoarabinomannan (LF-LAM) assay, Alere Determine TB LAM Ag (AlereLAM; Abbott, Chicago, IL, USA), is a simple and affordable point-of-care test that detects LAM by immunocapture. Despite low sensitivity, AlereLAM is recommended by WHO for TB diagnosis in severely ill or hospitalised HIV-infected adults, based on pragmatic field trials demonstrating its utility in reducing mortality through rapid detection and treatment initiation [39,40,41]. In addition to the AlereLAM, which is currently the only commercially available urine LAM test endorsed by WHO, the Fujifilm Silvamp LAM (FujiLAM) assay is being evaluated in a number of studies in children with HIV infection and SAM (severe acute malnutrition) [7]. A recent study in 204 children hospitalised with suspected TB in Cape Town, South Africa, showed similar sensitivities of FujiLAM and AlereLAM (42% and 50%, respectively), but lower than that of Xpert on sputum (74%). FujiLAM was more sensitive in children living with HIV (60%) and malnourished children (62%) and had higher specificity than AlereLAM (92% vs 66%, respectively) [42]. Emerging additional preliminary data from studies evaluating FujiLAM on frozen urine samples from children nonetheless suggests sensitivity and specificity, which do not meet the WHO target product profile for either a triage or confirmatory test [43].

### 2.5. Microbiological Tests for Drug Resistance

Drug resistance testing based on phenotypic (culture-based) or genotypic methods is needed to optimise the anti-TB antibiotic regimen according to the resistance pattern of the bacilli. Culture-based phenotypic drug-susceptibility testing (DST) methods provide a measure of the susceptibility of mycobacteria to an antibiotic [44]. DST was developed on solid media and is now usually performed on liquid culture media, with a shorter turnaround time. Genotypic tests predict resistance to a drug by detecting resistance-associated mutations rather than establishing in vitro susceptibility. Xpert can detect mutations in the rpo B gene involved in more than 95% of RIF resistance with a sensitivity of 99% and the Truenat MTB-RIF assay has shown a sensitivity of 84% in adults [45,46]. The WHO has also endorsed line probe assays (LPAs) which use different molecular technology to detect mutations associated with resistance [47]. The most widely studied/available tests are the GenoType MTBDRplus for rifampin and isoniazid, and the GenoType MTBDRsl-v1.0 assay (both from Hain LifeScience, Nehren, Germany) which identifies resistance to selected fluoroquinolones, aminoglycosides and ethambutol. LPAs are typically tested on positive culture isolates and are less sensitive if applied directly to raw specimens. Direct sputum testing by LPA is only recommended on smear-positive specimens, which are seldom relevant to paediatric TB.

Isoniazid and rifampicin resistance mutations can now also be detected on newly developed moderate complexity automated NAATs on high throughput platforms, such as the Abbott RealTime MTB RIF/INH, Bruker-Hain FluoroType MTBDR, Becton Dickinson Max MDR-TB assay, and Roche Cobas MTB RIF/INH assay. These fully automated tests for use in central laboratories are less complex and faster than phenotypic culture-based DST and LPAs [8]. With high daily test volume capacities, such platforms could be suited for large-scale programs in highly populated areas and good sample referral networks. Currently, no paediatric data are available on these platforms.

**Table 1:** Microbiological tests available for the diagnosis of intrathoracic tuberculosis in children.

	Principle	Limit of Detection (CFU/mL)	Delay to Positive Results (Negative If #)	Sensitivity in Children MRS (%)	Sensitivity in Children CRS (%)	WHO Recommendation	Type of Facility Where Usually Available in Resource-Limited Settings
Smear microscopy	Microscopic detection of MTB following Zeilh Neelsen or Auramine staining	1,000 to 10,000	Minutes	15–30	5–15	To be replaced as the initial diagnostic test by WRDs	Primary health centre with microscopy capacity
Solid culture	Phenotypic	10 to 100	3 to 4 weeks (8 weeks)	82	20–40	Monitoring of patient’s response to treatment.	National/regional level reference laboratory
Liquid culture	Phenotypic	<10	10 to 21 days (6 weeks)	85	20–40	Monitoring of patient’s response to treatment.	National/regional level reference laboratory
Xpert MTB/RIF **	Molecular detection of MTB and RIF resistance (rpoB gene) using GeneXpert system	131 112	2 h	62–66	25–35	Initial tests in children with signs and symptoms of pulmonary TB (strong recommendation)	Regional and district hospital *
Xpert MTB/RIF Ultra **	Molecular detection of MTB and RIF resistance (rpoB gene + IS6110) using GeneXpert system	38 15	90 min	64–75	45	Initial tests in children with signs and symptoms of pulmonary TB (strong recommendation)	Regional and district hospital *
TrueNAT **	MTB and RIF resistance detection using chip-based Real-Time (RT) micro-PCR on automated system	100	<1 h	No data in children	No data in children	Initial tests in children with signs and symptoms of pulmonary TB (conditional recommendation)	District hospital laboratory
Loop-Mediated Isothermal Amplification (LAMP)	MTB detection using amplification at a fixed temperature (without thermocycler) and simple visual detection	100	2 h	84 (1 study)	No data	Recommended only in adult as initial test so far	District hospital laboratory
Alere LAM	Detection of mycobacterial cell-wall glycolipid lipoarabinomannan in urine by immunocapture	No data	30 min	43 to 50 (HIV+)	No data	HIV-positive children with presumptive TB or advanced HIV disease or who are seriously ill or irrespective of TB suggestive signs if they have CD4 count <200 cells/mm^3^ (inpatients) or CD4 < 100 cells/mm^3^ (outpatients)	Point-of-Care, no need of laboratory.
FUJILAM Silvamp	Detection of mycobacterial cell-wall glycolipid lipoarabinomannan in urine by immunocapture	No data	60 min	42 to 65 (any children); 60 (HIV)	No data	Under review	Point-of-Care, no need of laboratory.

MRS: microbiological reference standard, CRS: clinical reference standard. References: [7,8,18,48,49,50]; * tested inside laboratories and primary health centres in ongoing studies; ** molecular WHO-recommended diagnostics = mWRD.

## 3. Microbiological Sample Collection Methods and Diagnostic Yield

In children, specimen collection methods, their feasibility and their ability to retrieve bacilli are key to achieving microbiological confirmation. Expectorated sputum, the recommended method for TB diagnosis in adults, is not feasible in children below 5 years of age, who are unable to expectorate, and remains challenging in children between the age of 5 and 10 years, in whom it can, however, be attempted with appropriate coaching and assistance [16].

Specimen collection methods recommended for children to replace expectorated sputum are gastric aspirate (GA) and induced sputum (IS), and more recently, nasopharyngeal aspirate (NPA) and stool (Table 2) [17,21,51]. Young children, as well as adults, swallow expectorations that may reach the oropharynx through a cough or mucociliary clearance, especially during the night. Given that MTB can survive the acidic environment of the stomach, GA is used to retrieve MTBc from bronchial secretions that have been swallowed. GA should be performed after overnight fasting and usually requires hospitalisation, although ambulatory GA has been shown to be effective [52,53].

The principle of IS collection is to induce cough and expectoration of bronchial secretions by nebulizing hypertonic saline. In younger children, it may also require mucus aspiration from the child’s nasopharynx through a cannula inserted in the nostril. IS provides a diagnostic yield equivalent to GA [21,54]. NPA relies on a similar nasopharynx aspiration procedure using a mucus trap connected to a suction device, without prior nebulization. NPA may achieve a similar yield as IS, although more data suggest that NPA yield is lower [55,56,57,58].

The use of stool specimens is based on the same concept as GA of retrieving MTB from swallowed respiratory secretions since MTBc travels through the gastrointestinal tract and is excreted in stool. Stool testing with Xpert has a sensitivity equivalent to that of respiratory samples in older children and those with extensive disease [59]. However, sensitivity in young children, who would most benefit from this sample collection strategy, is lower and may limit the stool’s utility as a specimen for diagnosis in this group [60,61]. Mycobacteria present in the stomach can also be retrieved for microbiological testing with the string test. Based on the Pediatric Entero-test® (HDC Corporation, Milpitas, CA, USA), this method enables to retrieve gastric fluid after 2 h of gastric downtime through a nylon string coiled in a capsule that is swallowed. The string test can only be performed in children aged >3 years and has a limited yield and potential as an alternative sample collection method [62].

Recent meta-analyses have gathered evidence on the diagnostic accuracy of mWRD Xpert and Ultra used on different specimens for diagnosis of intrathoracic TB against a microbiological reference standard; detailed results are presented in Table 3 [63]. Based on these results, WHO now recommends the collection of sputum or IS, GA, NPA or stool as specimens for both Xpert and Ultra testing [8,9]. Stool samples, however, require additional processing before Xpert testing as they contain PCR inhibitors, which can result in a high rate of invalid results, and debris/solid mass, which may increase error rates. Several methods for stool processing have been developed which include a centrifugation step and therefore require laboratories with adequate equipment and skilled staff. Three simplified, centrifuge-free stool processing methods, the FIND Stool Processing Kit (SPK), the KNCV Tuberculosis Foundation Simple One Step (SOS) method, and the TB-Speed Optimised Sucrose Flotation (OSF) method, are currently under evaluation in two head-to-head diagnostic accuracy and feasibility studies [64]. An interim joint analysis of these two studies has shown similar sensitivity between the three methods, but lower sensitivity than the microbiological reference standard, and high specificity with less than 10% of combined invalid and error results [64].

Finally, bronchoalveolar lavage (BAL), which consists in aspirating normal saline instilled in a subsegment of the lung through flexible bronchoscopy, has a diagnostic performance equivalent to GA in children with presumptive TB, with no or little added value when both techniques are done [23]. It has a place in children with severe intrathoracic TB who cannot be confirmed on less invasive specimens. BAL collection, however, requires highly skilled clinicians and equipment and remains beyond the reach of most low-resource settings.

### Operational and Safety Challenges for Implementing Paediatric Specimen Collection

There are several operational and safety challenges and considerations for the implementation of paediatric specimen collection procedures in resource-limited settings. All specimen collection procedures require specific technical training of healthcare workers, except for stool sample collection. IS requires equipment for the nebulization of the hypertonic solution, mucus aspirators to retrieve the specimen in young children and should be performed in a properly ventilated area to reduce the risk of MTB transmission from aerosolization during the procedure (Table 2). Due to the potential risk of bronchospasm caused by inhalation of the hypertonic solution, IS is contraindicated in children with respiratory distress. Mild nose bleeding resulting from the mucus extraction and vomiting are the most common adverse events reported with IS, followed by wheezing and transient hypoxia in <2% of children [65]. GA does not require specific equipment but does require consumables (e.g., syringes and nasogastric tubes) and minimal infection control and biosafety measures. To ensure a good quantity and quality of the sample and to avoid contamination of the aspirate with food or milk, GA should be done early in the morning, after overnight fasting, or after more than one hour in the supine position if done in an outpatient setting. NPA collection requires minimal infection control and biosafety measures compared to the collection of IS but also requires mucus extraction and aspirator, which can, however, be battery operated. First results from a large pragmatic TB diagnosis trial showed high feasibility of NPA collection among children with severe pneumonia in tertiary hospitals with 97% of children with successful NPA [66,67]. Although stool is a simple and non-invasive method, stool collection in outpatient settings, where it is often difficult to obtain a specimen on demand, may be a challenge. Results from the same trial show that it may also be challenging for inpatients, with only 81% of children with a stool collected as compared to 97% for NPA [66].

**Table 3:** Sensitivity and specificity of Xpert MTB/RIF and Xpert Ultra from different specimens in children with presumptive tuberculosis against the microbiological reference standard.

Type of Sample	Type of Test	Number of Participants	Sensitivity, % (95 CI)	Specificity, % (95 CI)
Expectorated or induced sputum	Xpert MTB/RIF	6812	64.6 (55.3 to 72.9)	99.0 (98.1 to 99.5)
	Xpert Ultra	697	72.8 (64.7 to 79.6)	97.5 (95.8 to 98.5)
Gastric aspirate	Xpert MTB/RIF	3487	73.0 (52.9 to 86.7)	98.1 (95.5 to 99.2)
	Xpert Ultra		64 (48 to 77)	95 (84 to 99)
Nasopharyngeal aspirate	Xpert MTB/RIF	1125	45.7 (27.6 to 65.1)	99.6 (98.9 to 99.8)
	Xpert Ultra	251	46 (29 to 63)	97.5 (94 to 99)
Stool	Xpert MTB/RIF	1592	61.5 (44.1 to 76.4)	98.5 (97.0 to 99.2)
	Xpert Ultra		53 (35 to 70)	98 (93 to 99)

MRS: microbiological reference standard based on culture from respiratory samples. Reference: [9,63,68,69].

## 4. Importance of Microbiological Diagnosis in Childhood TB

### 4.1. Reasons to Seek Microbiological Confirmation

Despite challenges in obtaining microbiological confirmation, the microbiological investigation remains very important for several reasons: (1) to allow rapid initiation of appropriate TB treatment, through the detection of MTBc and resistance to RIF and other TB drugs; (2) to assist in the diagnosis and management of complicated cases, especially when the differential may be broad; and (3) assess eligibility for the newly recommended 4-month regimen for treatment of non-severe presumed drug-susceptible tuberculosis: children with non-severe pulmonary TB based on CXR with either trace, very low or low semiquantitative Ultra results or negative smear microscopy results are eligible for the shorter regimen [9,68,69]. Furthermore, in research settings, optimising microbiological yield in order to obtain a robust reference standard is key in the evaluation of new diagnostic tools, or for assessing endpoints in vaccine or treatment studies. Xpert has notably been incorporated as a confirmatory test in diagnostic reference standards for research, at the same level as culture [70].

### 4.2. Variability in the Yield of Microbiological Testing in Children

The yield of microbiological detection in children depends on the pre-test probability of TB. A systematic review showed that the microbiological detection yield of Xpert ranged between 5% to 45% in studies with a low or high pre-test probability [65]. This probability varies with age and disease stage [6]. Intrathoracic lymph node disease is common in young children and most often paucibacillary unless complicated by bronchopneumonia, expansile lobar pneumonia, or cavitating Ghon focus. Adult-type pulmonary disease with infiltrates and cavities is more frequent in older children and is more likely to be microbiologically confirmed. Children identified with TB disease after household contact tracing often present with early paucibacillary forms of the disease. In this population, the yield of microbiological testing is very low [71]. On the other hand, children seen at tertiary referral or secondary health centres are more likely to present with advanced disease and are consequently more likely to be microbiologically confirmed compared to children seeking care at primary healthcare (PHC). As an example, in 384 children with presumptive TB enrolled at a PHC level in South Africa and who had 2 IS and 2 NPA performed, 26 (7%) and 30 (8%) tested positive by Xpert, and culture, respectively [57]. In another study implemented by the same group in tertiary referral hospitals using the same sampling strategy in 535 children, Xpert and culture were positive in 81 (15.1%) and 87 children (16.3%), respectively [19].

### 4.3. Specific TB Diagnostic Needs in Vulnerable Population Groups

Children with HIV infection and those with SAM are particularly vulnerable to TB as they are at higher risk than the general paediatric population of (1) developing TB disease when infected due to their cellular immune deficiency; (2) having undiagnosed TB as they may present with fewer and less specific symptoms; and (3) dying from TB, even when accessing TB treatment [72]. In a study of 262 antiretroviral therapy (ART)-naïve children living with HIV, Xpert positivity was associated with a 5-fold increase in mortality; TB treatment contributed to delayed ART initiation, which has serious consequences in children with low CD4 counts and advanced disease [73]. In this group, every effort should be made for prompt initiation of TB treatment, which requires optimised strategies for microbiological sample collection and testing, to enable early ART introduction. Data on the use of Xpert in children showed that there was no difference in test accuracy between HIV-infected and non-infected children [4]. Children with SAM often present with pauci-symptomatic forms of the disease and TB is often considered only in case of persistent wasting despite therapeutic feeding, exposing those with severe disease to a high risk of death. In children with SAM who are hospitalised, more intensive microbiological sampling and testing for potential underlying TB are needed. In children with SAM or HIV, LAM assays should be part of the diagnostic package despite their limited sensitivity, as they are more likely to detect disseminated forms of TB that are more frequent in these vulnerable groups [42,74,75,76].

Children with severe pneumonia are at a high risk of mortality and have a prevalence of microbiologically confirmed TB between 5.9% and 7.5%, with an inter-country variability and higher prevalence in HIV-infected children [77,78,79]. Most children with TB-associated pneumonia present with acute symptoms and are often not initially investigated for TB. Recent results from a cluster-randomised trial in young children admitted with severe pneumonia showed that the combination of NPA and stool samples tested with Ultra was highly feasible and led to microbiological confirmation in 28% of children diagnosed with TB [80]. This study confirmed the importance of TB as an aetiology in severe pneumonia.

Finally, children not improving on treatment are at higher risk of drug-resistant TB and would benefit from appropriate and repeated microbiological testing, including DST.

## 5. Challenges in Implementation and Access to Microbiological Diagnosis in Children

Globally, children have poor access to appropriate TB diagnostic tools because they are often not screened or tested for TB when they do access clinics. In an observational study conducted in 13 county hospitals in Kenya between 2016 and 2018, among 23,741 children with ≥2 signs and symptoms suggestive of TB, 62% were thoroughly screened for TB and received a full physical examination, but TB tests were performed in only 392 (1.7%) [81]. Sub-optimal childhood TB diagnosis is also explained by poor availability of appropriate microbiological sample collection and testing, regardless of the level of healthcare. In 2010, from 651 sites providing HIV care in 9 African countries, 87% had smear microscopy but only 6% and 5% of those sites had the capacity to collect IS and GA, respectively [82]. In a cross-sectional survey conducted in 2018 to assess childhood TB diagnosis capacity across 179 PHCs in 32 districts of Cambodia, Cameroon, Côte d’Ivoire, Mozambique and Uganda, specific sample collection for children was available in 6.7% of the PCHs and only 46% had an onsite laboratory [83]. The poor uptake of microbiological tests in children may also be explained by their poor sensitivity: sample collection methods may be perceived by health care workers as challenging and burdensome in young children, especially if/when they only poorly contribute to the diagnosis overall. Beyond childhood TB, mWRD rapid molecular tests were only used as the initial diagnostic test in 33% of new TB cases overall in 2020, highlighting the lack of access to these more sensitive diagnostic tools in many healthcare settings [1].

One of the barriers to accessing microbiological testing may reside in the acceptability, feasibility and implementation challenges of different childhood TB sampling and testing methods in different settings. Initial findings from the TB-Speed project in six high-incidence settings from sub-Saharan Africa and South-East Asia showed that NPA collection, using a battery-operated mucus aspirator system and stool collection was feasible and well-accepted, regardless of the level of healthcare pyramid (tertiary hospitals, district hospitals, primary care clinics), both in the general paediatric population and in children with severe pneumonia, HIV and/or SAM. NPA sample collection most often required the involvement of two people at least (two health care workers (HCW) or an HCW plus parent or colleague). Mixed feelings about NPA were shared by parents and HCWs, including pain/discomfort/distress of the child during NPA, which can be expected, but none superseded the overall acceptability and feasibility, including in children with severe pneumonia [67]. Sub-optimal feasibility and tolerability of NPA were recently reported in another prospective cohort of HIV-infected children followed-up at a tertiary hospital in Burkina Faso [84]. Data available so far suggest that stool collection is perceived as easy and adaptable to all children. However, stool sampling may raise feasibility issues, as it is frequently not obtained during the child’s presence at the facility (or even later due to transport and financial constraints for parents to travel back with a stool container) [67]. The main factors contributing to the acceptability of NPA and stool sampling seem to be valuing child health benefits (over any possible constraint), parents being well informed and supported, as well as skills among nurses for informing/motivating parents and obtaining results quicker [67]. In the TB-Speed Decentralisation study, Ultra testing of NPA using a battery-operated G1 Edge platform was implemented by nurses in 24 PHCs and several tertiary level hospitals. Ultra testing was not reported as technically difficult to implement by nurses [67]. Stools were collected at PHCs and referred to a district hospital for laboratory-based testing, but poor sample transport conditions between facilities (long distances, bad terrain, heavy rains and challenges in maintaining storage temperature) and power instability both at the district hospital and PHC levels were common challenges, limiting the feasibility of stool-based testing approaches.

## 6. Strategies to Improve Yield and Access to Microbiological Diagnosis

### 6.1. Combining Samples

The collection and testing of multiple samples may contribute to an increased diagnostic yield and improved diagnosis. The former WHO TB DOTS strategy relied on repeated sputum smears. Access to more sensitive molecular tests provides an opportunity to reduce the number of tests needed to accurately diagnose TB. WHO currently recommends to repeat microbiological samples and testing on either sputum, GA, NPA or stool specimens in children only when the pre-test probability, i.e., the prevalence of culture-confirmed TB in the group/settings is ≥5%. However, this was a conditional recommendation, with a low certainty of evidence in test accuracy for sputum and very low for other specimens [8]. In a study of 300 children with presumptive TB aged <5 years in Kisumu, Kenya, testing a second sample of the same kind (GA, NPA, or IS) led to an average incremental yield of 8% to 10% by Xpert or MGIT, respectively [85]. A systematic review reported that testing a second sample contributes between 6% and 33% of the cumulative microbiological yield using Xpert or culture, regardless of the sample collection method [65]. Collecting samples of different types, particularly including samples that are less invasive and easier to implement from a technical point of view, may significantly contribute to increasing the yield of microbiological testing. In the same study of children with presumptive TB in Kenya, combining one NPA and one stool increased the sensitivity to 71% compared to 66% for one NPA specimen, with results similar to what was obtained using two GA or two sputum samples [85]. Similarly, in another international diagnostic accuracy study in HIV-positive children with presumptive TB from four high-incidence countries, Xpert testing of one NPA and one stool increased sensitivity from 69% for NPA only and 62% for stool only, to 75% when using both [62]. A single study has evaluated pooling (mixing together in the laboratory) samples of different types (GA, IS, and NPA) in order to increase the bacillary concentration in the pooled sample. The study concluded that the diagnostic yield of a pooled GA/IS/NPA sample by Xpert and culture was equivalent to that of a single GA sample, which remains clearly a more feasible and less resource-intensive strategy [56].

### 6.2. Improving Access to Sample Collection and Testing at Lower Levels of Health Care

Sample referral and hub systems may be an efficient option to improve access to sample collection at lower levels of healthcare while maintaining centralised laboratory systems with higher throughput and more experienced staff. Countries such as Uganda have made this choice [50]. As the GeneXpert platform also enables testing for other pathogens, such as HIV, hepatitis B or C viruses, SARS-CoV-2, Ebola, and Human Papilloma Viruses, it may be a promising strategy to have such devices and capacity at the district hospital level. The centralised approach can be challenged by poor sample transport conditions between facilities.

Another approach to improve access to childhood TB diagnosis is to decentralise sample collection and testing using mWRDs at lower levels of the health system. Both NPA and stool specimen collection have the potential to be implemented in outpatient settings, with limited infrastructure and equipment, minimal infection control and biosafety measures, and no need for the child to fast before the sample collection. For stool, the upcoming simplified sample processing methods are expected to facilitate Ultra testing at the PHC level. Furthermore, the battery-operated G1 Edge GeneXpert devices enable molecular testing at low levels of healthcare. New and even simpler sample methods, such as oral swabs, may also be of interest for implementation at a low level of healthcare but appear to lack sensitivity [86]. Decentralisation of childhood TB services is now recommended by WHO, but evidence on the impact on morbidity, mortality, treatment outcome and cost-effectiveness of fully decentralising microbiological diagnosis at the PHC level is still lacking. Furthermore, although promising, the decentralisation of child-adapted sample collection and molecular diagnosis at the PHC level can be burdensome and therefore requires the careful thinking of work organisation and priorities for PHC staff who are usually already very busy with dealing with all medical needs and facility management activities. Quality assurance and close supervision through the national TB reference laboratory and clinical mentoring for case management in those with negative microbiological results will be key to such a strategy.

### 6.3. Improving Screening of Children with Presumptive TB

Screening plays a key role in identifying children with presumptive TB for full diagnostic work-up. Screening strategies have been recommended by the WHO for several adult target populations, for children living with HIV or household child contacts based on simple symptom questionnaires or chest X-rays, but not systematically for sick children at health facilities. In the newly released WHO-recommended algorithms for childhood TB, screening for presumptive TB should be integrated into the routine assessment for childhood diseases using the Integrated Management of Childhood Illnesses approach [69]. Whether standalone screening for TB at triage for the upfront identification of children with presumptive TB for further testing will positively impact TB detection and the yield of microbiological tests, is currently under evaluation in the TB-Speed project. The impact of screening on microbiological confirmation and on patient-important outcomes may be substantial in specific high-risk groups who have a higher pre-test probability of TB disease but a less typical presentation (such as children with severe acute malnutrition and acute pneumonia from high burden settings) [77,87]. Specific recommendations for targeted screening of these high-risk groups are not yet in place. Conversely, the microbiological yield is expected to be lower among screened children with early forms of the disease who are more likely to be diagnosed clinically or radiologically [8,87] Interferon-gamma release assays (IGRAs) have a prominent role as screening tests for TB infection in high resource settings, for children from epidemiologically high-risk groups. However, they cannot distinguish latent from active TB. In addition, high cost has restricted their uptake in resource-limited, high TB burden settings.

### 6.4. New Biomarkers for Microbiological Diagnosis

Improvement of microbiological detection requires the development of new biomarkers. Technologies using nanoparticles, which are small particles that can be bound to biomolecules for diagnostics in cancer and infectious diseases, have notably shown promising results with the diagnosis of TB [88]. Nanotechnologies have been recently used to develop tests to detect MTB or antituberculosis drug resistance mutations with either PCR or lateral flow strip technologies, with performances surpassing that of Xpert or other standard microbiological tests. [89,90]. Although not the focus of this manuscript, biomarker development also targets specific host immune responses related to TB disease. Transcriptomic detection of host RNA expression may have the potential to distinguish between TB infection and disease, which is not currently possible with interferon-gamma release assays or tuberculin skin testing [91]. Performance of first gene expression signatures on a large number of genes had limited but promising performance for the diagnosis of childhood TB, notably in those without microbiological confirmation [92]. Whether newer signatures with a reduced number of genes, such as those proposed to predict progression to TB in recent household contacts, will play a role in routine childhood TB diagnosis resource-limited settings warrants further research [93].

## 7. Conclusions

The field of microbiological TB diagnosis in children is a rapidly evolving landscape. WHO recently made recommendations towards more child-friendly, simple, affordable sample collection methods that can be implemented at lower levels of healthcare. In addition, new mWRD, including Xpert and Ultra, contribute to bringing diagnostic performances close to that of culture to primary health care level, with short turnaround times. Several pending research gaps remain, however, these are related notably to clinical utility and the cost-effectiveness of microbiological testing that still hampers its wide-scale adoption and deployment by national TB programs. The WHO recommendation to further decentralise childhood TB diagnostic services is both a major opportunity to fill in the gaps in access to appropriate sample collection and testing for children at the PHC level and a call for urgently needed implementation and operational research.

Despite increasing access to molecular testing, microbiological diagnosis still has major limitations and cannot be used as a rule-out test. Even in settings where there is good uptake of diagnostics, most or a large proportion of diagnosis is and will still be clinical, thus emphasising the need for skills strengthening and adequate comprehensive diagnostic algorithms [94]. In this context, among the most important steps to improve microbiological diagnosis and contribution in children will be to (1) develop triage tests for referral, as advocated by the WHO TPP; (2) develop more sensitive non sputum-based and point-of-care tests; (3) specifically target high-risk groups, including children with HIV, SAM and severe pneumonia; and (4) assess/refine the role of microbiological diagnosis as the first step in diagnostic algorithms following recent WHO recommendations (Box 1) [68].

Box 1 Pending research gaps for the microbiological diagnosis of childhood TB.

- **Performance of tests and sample collection methods**
  ◦ Diagnostic yield of multiple specimens of different types versus repeated specimens and incremental yield of different combinations of specimen types.
  ◦ Diagnostic performance of combining bio-signatures/omics approaches with traditional microbiological approaches.
  ◦ Feasibility and impact of methods to enhance the viability and growth of bacilli from culture on overall microbiological yield.
  ◦ Diagnostic accuracy and clinical impact of LAM assays in severely malnourished children.
  ◦ New approaches to simplify sample collection, such as the oral swab.
- **Placement of tests in paediatric diagnostic algorithms**
  ◦ Triage or screening tests to identify children with presumptive TB and target groups at higher risk of TB for sample collection, regardless of symptoms.
  ◦ Role and contribution of microbiological diagnosis in treatment decision algorithms.
  ◦ Clinical utility of Xpert testing in specific groups to reduce delay to treatment initiation.
- **Implementation of tests**
  ◦ Feasibility, acceptability, impact on morbidity, mortality, treatment outcome, cost-effectiveness, and budget impact/cost of decentralising childhood TB microbiological diagnosis at PHC level.
- **New biomarkers**
  ◦ Development of more sensitive and specific biomarkers of active disease, given the limitations of microbiological diagnosis.

## Figures and Tables

**Table 2:** Microbiological specimen collection methods for diagnosis of intrathoracic tuberculosis in children.

Type of Sample	Principle	Age Group	Time to Obtain Specimen	Specific Requirements	Equipment and Consumables Needed	Biosafety and Infection Control Risk Assessment	Patient Safety Concern
Expectorated sputum	Collect spontaneously expectorated sputum	Older children and adolescents	Seconds to minutes		Sputum containers	Low	No safety concern
Induced sputum	Induce sputum through nebulisation of hypertonic saline	All	Several minutes	Adequate infection control measures due to aerosolisation of secretions	Nebulization machine, hypertonic saline solution + suction in young children (see NPA below)	Moderate (need a well-ventilated area due to risk of aerosol)	Moderate (contraindicated for children with respiratory distress)
Nasopharyngeal aspirate	Aspirate 2 mL of expectoration in retropharynx	All	Seconds (12 s per nostril)	Supine or seated position; caregiver or HCW to help restraining Induces reflex cough; saline instillation feasible	Suction machine (low negative pressure needed 80 to 100 mmHg) and mucus aspirator	Low	Very low
Gastric aspirate	Aspirate 5 to 10 mL of gastric content	<5 to 8 years	Minutes	Overnight fasting, hospitalisation	Syringe and nasogastric tube	Low	Very low
Stool	Collection of stool for detection of swallowed sputum	All; acceptability for older children may be reduced	Time to pass stools	Storage and transport; Processing before Xpert testing is not standardised; data suggest stool detects TB in children with high bacillary loads	Plastic container	Very low	No safety concern
String tests	Collection of swallowed sputum by an absorbent string coiled into a capsule and swallowed into the stomach	>4 years	2 h	Not widely adopted	Entero-test (capsule containing lead weight and string)	Low	Very low

MRS: microbiological reference standard based on culture from respiratory samples.
